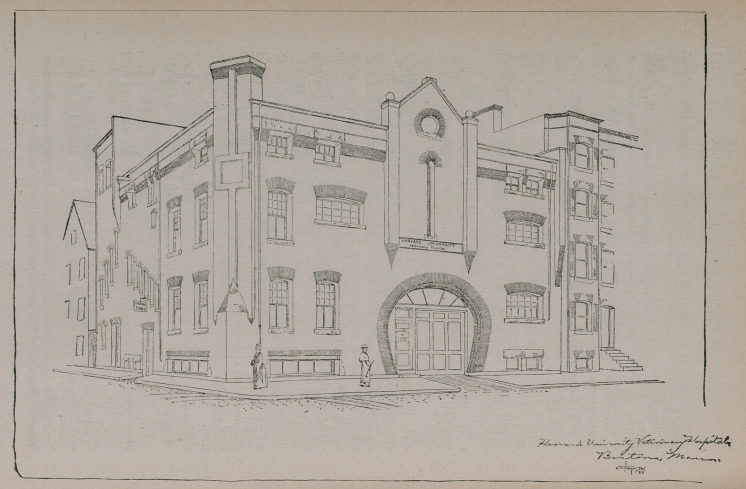# The Harvard Veterinary School

**Published:** 1888-04

**Authors:** 


### ART. XIV.—THE HARVARD VETERINARY SCHOOL.

In the year 1882 the Faculty of the Harvard Medical School, appreciating the importance of comparative medicine, recommended to the officers of the Harvard University the establishment of a veterinary school. Accordingly in September, of that year, Charles P. Lyman, F.R.C.V.S., was appointed by the University Professor of Veterinary Medicine.

The organization of the school was at once undertaken, and in November arrangements were made for the building of a suitable hospital, where cases could be received and shown to the students. With the assistance of the Massachusetts Society for Promoting Agriculture, of Dr. William F. Whitney and Mr. Henry L. Higginson, money was obtained to buy a lot of land, centrally located in the city of Boston and to erect thereon a substantial and convenient building, capable of accommodating twenty-three horses and fifteen dogs; with rooms and offices for the officers and servants of the hospital. A shoeing-forge was also built in the cellar.

This building has since been enlarged and can now accommodate thirty-four horses and thirty dogs, and has also had added to it a large lecture room, museum, reading and dissecting rooms.

In September, 1883, the first class, consisting of nine students, entered the school. To enter the school it is nec-

Harvard University Veterinary Hospital.

Boston, Mass.

H. W. Thayer Del '88

essary to pass an entrance examination in Spelling, Dictation, Reading, Arithmetic and Composition. Latin, French, German and Algebra are not compulsory, but are taken by some of the students.

The course covers three terms of nine months each, extending from the last Wednesday in September to July.

Throughout the first year the course in Physiology consists of two lectures and two recitations every week. These are given by Professor Henry P. Bowditch, and are accompanied by numerous experiments on animals by scientific apparatus, and are illustrated by plates. Students are also given opportunities to perfoım a series of experiments personally, in the Laboratory.

Assistant Professor William B. Hills gives three exercises a week on Chemistry; all illustrated by practical experiments. Each student is provided with a desk and apparatus in the Laboratory, and is required to do a prescribed amount of work in practical and analytical chemistry. Two examinations are held on this subject, one on theoretical, chemistry, and in the other a compound is given to each student, which he is required to analyse.

Practical Histology is taught by Drs. Henry P. Quincy and Charles S. Minot. Four exercises a week are given, and the minute anatomy of the various tissues of the body explained and demonstrated by the use of the microscope. Students provided with microscopes are given facilities for original work and are taught, how to make, stain and mount preparations and sections.

In the last part of the first year lectures are given on Hygiene and Embryology. The course on Hygiene, besides dealing with the various points on sanitation, treats of the adulteration of foods, especially of butter and milk; this last being of great importance to the veterinarian. Eight lectures on the development of the embryo comprise the course on embryology.

Three lectures a week are given in Comparative Anatomy by Dr. Daniel D. Lee, and these lectures are illustrated by specimens from the museum, plates and pápière maché models of the various parts.

From November to April the dissecting room is open, and each student is required to dissect all parts of the body, either on horses, dogs or cattle, under the direction of the demonstrators and Dr. Lee.

Dr. Kenelm Winslow gives two lectures a week on Botany in the last half of the year, paying particular attention to the most valuable forage plants and to the more common poisonous ones.

Beginning in January Professor Lyman gives one lecture a week on Form and Action of the Horse, explaining the various unsoundnesses of horses, and showing by practical demonstration how they should be examined for soundness.

At the end of the first year students are required to pass examinations in the studies of the course and to present a certificate of dissection.

If a student successfully passes any two of the studies he is advanced to the second year. During the summer recess, students are allowed to attend the hospital and there gain much practical knowledge.

Throughout the second year the lectures on the Theory and Practice of Veterinary Medicine, are given by Professor Lyman, three times weekly and extend over two years, being taken by both the second and third year students. In this course all the diseases of the different animals are taken up and considered as to their treatment, diagnosis, prognosis, etiology and the connection which they may have with diseases of man. Pathological specimens from all the domestic animals are frequently examined at the hospital, and these are used by Professor Lyman to illustrate the post mortem appearances in the different diseases.

The clinical facilities of the school are of the best and are open to the second and third year students. The Professors hold clinics in the hospital and in their private practice.

Two lectures weekly for the first half of the second year are given by Dr. Kenelm Winslow, on Materia Medica, and the students are made familiar with the compounding of drugs by one exercise in practical pharmacy weekly.

Professor Reginald H. Fitz, gives two lectures and two recitations a week on Pathological Anatomy. At the recitations, morbid specimens are exhibited to the students and conferences held on them. The lectures are illustrated by specimens from the collection of the Warren Museum. In addition two exercises weekly are given in Pathological Histology ; the students examining morbid specimens with the microscope, and diagnosing the lesions under the direction of Dr. Whitney and Dr. Gannett.

Medical Chemistry which treats of the chemical analysis of the urine and the microscopical examination of the sediment is taught by Professor Edward S. Wood. In the last half of the year, Professor Wood delivers a course on Toxicology, explaining the symptoms and post-mortem appearances, resulting from the various poisons, and the treatment of such cases. In this course each student has a desk in the chemical laboratory, where he is required to make examinations of urine, both chemical and microscopical and so to diagnose the various diseases of the urinary tract. Analysis of compounds containing the more common poisons is also required.

Dr. Harold C. Ernest, gives a series of lectures on Bacteriology, and students desiring to investigate this interesting subject can enter the laboratory, which is furnished with all the necessary apparatus.

To enter the third year the student must pass *all* the examinations of the first year, and the majority of those of the second.

In the third year, Dr. William F. Whitney gives one lecture a week, in the first half on Parasitic Diseases, and in the second half Professor Lyman takes this hour for his lecture on Obstetrics.

Dr. Frederick E. Cheney, gives a course on Ophthalmology, and exercises on the use of the ophthalmoscope.

A course of lectures on Operative Veterinary Surgery, is given by Dr. Daniel D. Lee, and third year students are required to perform all of the more common operations at exercises given for that purpose.

Three lectures on Therapeutics are given weekly, by Assistant Professor Francis H. Williams, and numerous demonstrations made of the action of drugs on living animals.

Professor David W. Cheever and Assistant Professor J. Collins Warren, give a course on the Theory of Surgery.

Members of the third class have opportunities of going to the Brighton Abattoir, where they are instructed in the inspection of meat. All spare time is spent in the hospital, or in out visits. Two of the members of the third class are appointed House Surgeons, and all the other members of the class are required to serve in turn as Dressers and Dispensers under the direction of Dr. Edward C. Beckett, Superintendent of the Hospital.

At the present time there are twenty-eight students at the School.

To obtain the University Degree, *Medicinæ Doctoris Veterinariæ*, the student must pass all examinations, and be twenty-one years of age. Up to the present time, the School has graduated two classes.

Endowments are much needed for the support and improvement of the School, no part of the funds belonging to the University being available, as they are all appropriated to specified purposes.

## Figures and Tables

**Figure f1:**